# Efficient diagnosis of psoriasis and lichen planus cutaneous diseases using deep learning approach

**DOI:** 10.1038/s41598-024-60526-4

**Published:** 2024-04-27

**Authors:** Arshia Eskandari, Mahkame Sharbatdar

**Affiliations:** Faculty of Mechanical Engineering, K. N. Toosi University of Technology, Tehran, Iran

**Keywords:** Deep learning in dermatology, Lichen planus, Psoriasis, Diagnosis, ResNet-50 model, Skin diseases, Biomedical engineering

## Abstract

The tendency of skin diseases to manifest in a unique and yet similar appearance, absence of enough competent dermatologists, and urgency of diagnosis and classification on time and accurately, makes the need of machine aided diagnosis blatant. This study is conducted with the purpose of broadening the research in skin disease diagnosis with computer by traversing the capabilities of deep Learning algorithms to classify two skin diseases noticeably close in appearance, Psoriasis and Lichen Planus. The resemblance between these two skin diseases is striking, often resulting in their classification within the same category. Despite this, there is a dearth of research focusing specifically on these diseases. A customized 50 layers ResNet-50 architecture of convolutional neural network is used and the results are validated through fivefold cross-validation, threefold cross-validation, and random split. By utilizing advanced data augmentation and class balancing techniques, the diversity of the dataset has increased, and the dataset imbalance has been minimized. ResNet-50 has achieved an accuracy of 89.07%, sensitivity of 86.46%, and specificity of 86.02%. With their promising results, these algorithms make the potential of machine aided diagnosis clear. Deep Learning algorithms could provide assistance to physicians and dermatologists by classification of skin diseases, with similar appearance, in real-time.

## Introduction

The accurate diagnosis and effective treatment of a dermatological ailment within the domain of dermatology are notably contingent upon the morphological attributes and visual presentation of diverse cutaneous lesions. The diagnostic procedure encompassing dermatoses and skin disorders necessitates the assimilation of multifarious data points, including the patient's medical background, clinical manifestations, dermatological imagery, and periodic histopathological assessments conducted by a seasoned dermatologist. Nonetheless, given the profusion of skin maladies and their perceptible similarities, relying solely on human observation oftentimes proves insufficient for achieving precise diagnostic outcomes. Consequently, this predicament engenders perplexity in attaining an unequivocal diagnosis^1–4^. Misdiagnosis has been documented in approximately 32% of instances involving such maladies^5^.

An exemplification of diseases possessing a remarkable resemblance to one another, engendering frequent confusion, can be discerned in psoriasis and lichen planus skin ailments. Psoriasis, an endemic and persistent cutaneous ailment, manifests as erythematous patches and desquamating skin across diverse bodily regions, such as the elbows and knees. Despite its prevalence, a comprehensive understanding of its therapeutic modalities remains elusive. Notably, psoriasis exhibits a cyclic proclivity, featuring episodic occurrences on a weekly or monthly basis^6,7^.

Being a chronic inflammatory dermal disorder influenced by genetic factors, immune dysregulation, and periods of exacerbation, psoriasis afflicts approximately 1–3% of the global populace. While clinical assessment forms the basis for its diagnosis, histopathological examination of a cutaneous biopsy specimen bestows more comprehensive findings. Although psoriasis seldom directly threatens the patient's life, it imparts considerable incapacitation and is accompanied by underappreciated societal and economic ramifications^8^. Conversely, lichen planus, another prevalent skin pathology, engenders inflammatory reactions, irritation, and edematous manifestations, presenting lesions that closely resemble psoriasis. Both conditions bear an association with the intricate workings of the immune system. It is pertinent to note that lichen planus eludes a definitive remedy, though pharmacological interventions and therapeutic measures hold promise in expediting convalescence and assuaging discomfort^9,10^. Psoriasis, an ailment with multifactorial origins, exhibits a plethora of manifestations, extending beyond the confines of the skin. As of present, no precise diagnostic criteria exist to discern psoriasis. Instead, the diagnosis and classification of this malady rely upon diverse clinical phenotypes, age of onset, severity of symptoms, and morphological attributes of the disease. Despite the expansive clinical spectrum accompanying this condition, only a well-trained and experienced physician possesses the proficiency to accurately diagnose psoriasis and its distinct phenotypes^11^. Seasoned physicians can often make a clinical diagnosis of lichen planus based on its characteristic presentation. Nevertheless, for atypical cases, a 4-mm punch biopsy becomes imperative^12^. As per Van Der Meij et al., certain phenotypes of this disorder may even render biopsies unhelpful, providing incomplete information to the attending physician^13^.

As previously noted, distinguishing between psoriasis and lichen planus poses a significant challenge due to their similar presentations compared to any other skin lesion. Consequently, numerous studies spanning from 2000 to recent years^14–17^ have explored various lesion characteristics and diagnostic methods to enhance accuracy in detection of these diseases. Despite advancements highlighted in these investigations, accurately identifying these conditions still necessitates dermoscopic imaging, a process requiring a clinic visit and considerable time. Furthermore, even with such imaging, disease classification remains somewhat unreliable.

Artificial Intelligence (AI) is an expansive concept that denotes the utilization of computational systems to emulate intelligent conduct while minimizing reliance on human intervention. The utilization of artificial intelligence in the realm of detection and categorization holds paramount significance. Consequently, psoriasis and lichen planus, two dermatological afflictions, present noteworthy prospects for applying artificial intelligence techniques.

An expanding body of scholarly literature revolves around the realm of skin disease detection. Countless researchers have diligently delved into the realm of machine learning and deep learning techniques, striving to unravel novel and enhanced avenues for identifying such ailments through the analysis of images.

A scientific inquiry conducted by Yang et al. pursued the training of an adept deep-learning network to discern dermoscopic images of psoriasis and other papulosquamous diseases. The ultimate aim was to enhance the precision of psoriasis diagnosis. The study entailed harnessing the prowess of the EfficientNet-B4 architecture, which underwent training on a corpus of 7033 dermoscopic images derived from 1166 patients, gathered from the esteemed Department of Dermatology at the Peking Union Medical College Hospital in China. A five-fold cross-validation was executed on the training set to evaluate the efficacy of EfficientNet-B4 against other prevalent networks utilized in prior investigations. Subsequently, a test set of 90 images was employed to draw a comparison between our four-class model and the diagnostic acumen of board-certified dermatologists. Pertinent information, encompassing the age and professional titles of dermatologists, was gleaned through an online questionnaire. Notably, the psoriasis-specific two-classification and four-classification models established in the study demonstrated remarkable precision in discriminating among various papulosquamous skin diseases. Their performances displayed striking parity with the average expertise of experienced dermatologists. Consequently, these models present formidable support for augmenting the diagnostic process of psoriasis^18^.

In a similar research, Zhao et al. endeavored to devise a psoriasis identification system grounded in clinical images, void of reliance on a dermatoscopy, while mirroring the effectiveness of a dermatologist. Their approach entailed the exploration and comparison of an assemblage of deep learning models, employing convolutional neural networks (CNNs), to achieve automatic psoriasis identification. The researchers conducted their investigation on an extensively curated dermatological dataset comprising 8021 clinical images capturing 9 prevalent disorders, including psoriasis, alongside comprehensive electronic medical records spanning a prodigious 9-year period in China. A two-stage deep neural network was generated for the purpose of psoriasis detection. In the initial stage, a multilabel classifier underwent rigorous training to discern the distinctive visual patterns exhibited by each distinct skin ailment. Subsequently, in the second stage, the outcomes of the first stage were effectively harnessed to discriminate psoriasis from other skin diseases^19^.

In a study by Zhu et al., a novel deep-learning framework was constructed and trained on a dataset representing the real clinical environment of a tertiary hospital in China. The dataset consisted of 13,603 dermatologist-labeled dermoscopic images, encompassing 14 disease categories. The authors concluded that their retrained framework accurately classified common dermatoses encountered in outpatient practice, including infectious and inflammatory dermatoses, as well as benign and malignant cutaneous tumors^1^.

Another investigation conducted by Bajwa et al. sought to expand upon prior research in Computer-Aided Diagnosis within the field of dermatology by delving into the prospective applications of Deep Learning in the classification of numerous skin diseases. The objective was to enhance the performance of classification and exploit disease taxonomy. In order to achieve this, cutting-edge Deep Neural Networks were trained using two of the most extensive publicly accessible skin image datasets, specifically DermNet and ISIC Archive. Additionally, disease taxonomy was utilized whenever available to augment the classification performance of these models. This study exemplifies the tremendous potential of Deep Learning in accurately categorizing a wide spectrum of skin diseases, achieving levels of accuracy comparable to human performance while also surpassing it in terms of reproducibility. Consequently, Deep Learning holds considerable promise in the realm of practical real-time skin disease diagnosis, as it can assist physicians in conducting large-scale screenings using clinical or dermoscopic images^20^.

In a parallel study conducted by Gunwant et al. in 2022, an advanced expert system was developed utilizing the EfficientNet B-0 and ResNet-50 models. The primary objective of this system was to support clinicians in accurately identifying various cutaneous diseases, including Eczema, Psoriasis, Lichen Planus, Benign Tumours, Fungal Infections, and Viral Infections. Similar to prior research endeavors, the study leveraged the DermNet dataset for comprehensive analysis. Moreover, in line with conventional methodologies, the study grouped Psoriasis and Lichen together for identification purposes. The results yielded an impressive average accuracy rate of 91.36%, highlighting the effectiveness and reliability of the proposed approach^21^.

In 2023, Mohamed Hammad et al. introduced "Derma Care," a deep learning approach aimed at detecting eczema and psoriasis skin conditions. Leveraging a publicly available image dataset from Kaggle^22^, comprising 27,153 images categorized into ten classes of skin diseases, the study concentrated on two specific classes: 1677 images for eczema and 2055 images for psoriasis. The researchers evaluated three prominent deep learning algorithms—AlexNet, ResNet, and VGG-16—and achieved impressive results, boasting an accuracy of 96.20%, precision of 96%, recall of 95.70%, and F1-score of 95.80%. Similar to related scholarly investigations, this study also has only focused on Psoriasis condition^23^.

Nieniewski et al. in 2023 conducted a study focusing on differentiating Psoriasis from other dermatoses using a small dataset and transfer learning. The VGG16 deep convolutional neural network is used as a feature extractor, and the Support Vector Machine classifier is used for classification. The study uses a small number of 75 Psoriasis patients and 75 non-Psoriasis patients, with a variable number of clinical images taken for each patient. The input images are obtained with smartphone cameras without any special arrangements or equipment, leading to variability in working conditions. Their method achieved the sensitivity of 85.33% and a precision of 82.58%. Similar to many other investigations the paper only has focused on Psoriasis^24^.

In the aforementioned investigations, the primary emphasis of the authors predominantly revolved around Psoriasis dermatoses, whereas the latter examination did not make any distinction whatsoever between Psoriasis and Lichen planus. In the inquiry conducted by Zhu et al., it was articulated that the primary objective of the study entailed the establishment of an innovative framework, one that authentically replicated the clinical milieu prevailing in China. This endeavor aimed to enhance the applicability of artificial intelligence (AI) within clinical practice for the benefit of Asian patients. Consequently, the outcomes of this study possess an excessive degree of specificity, thereby rendering them unsuitable for extrapolation to alternative clinical settings. Moreover, none of the investigations have undertaken a direct comparative analysis between these two skin disorders in the absence of concurrent ailments. The resemblance between Lichen Planus and Psoriasis skin diseases is considerable, often leading to their classification within the same category. While expanding the number of classes may enhance overall classification accuracy, this could potentially diminish the model's validity for detecting these specific lesions. Given the study's focus on these two skin conditions, prioritizing their accurate identification outweighs the pursuit of inflated overall accuracy, hence only these two lesions were considered for this investigation. Given the conspicuously high level of resemblance between these two dermatoses, it is imperative to explore these lesions independently from other skin conditions in order to produce reliable findings that can be effectively implemented in an authentic clinical environment.

## Materials and methods

This segment elucidates the methodologies and constituents employed in this study. The initial division presents a comprehensive exposition of the dataset and its corresponding classes, whereas "Class Balancing and Data Augmentation" section concentrates on the harmonization of classes and the amplification of data. Subsequently, "Data Pre-Processing" section offers a succinct account of the preprocessing procedure. Ultimately, the expounded ResNet-50 model is explicated in "ResNet-50" section.

### Dataset

**Figure 1:**
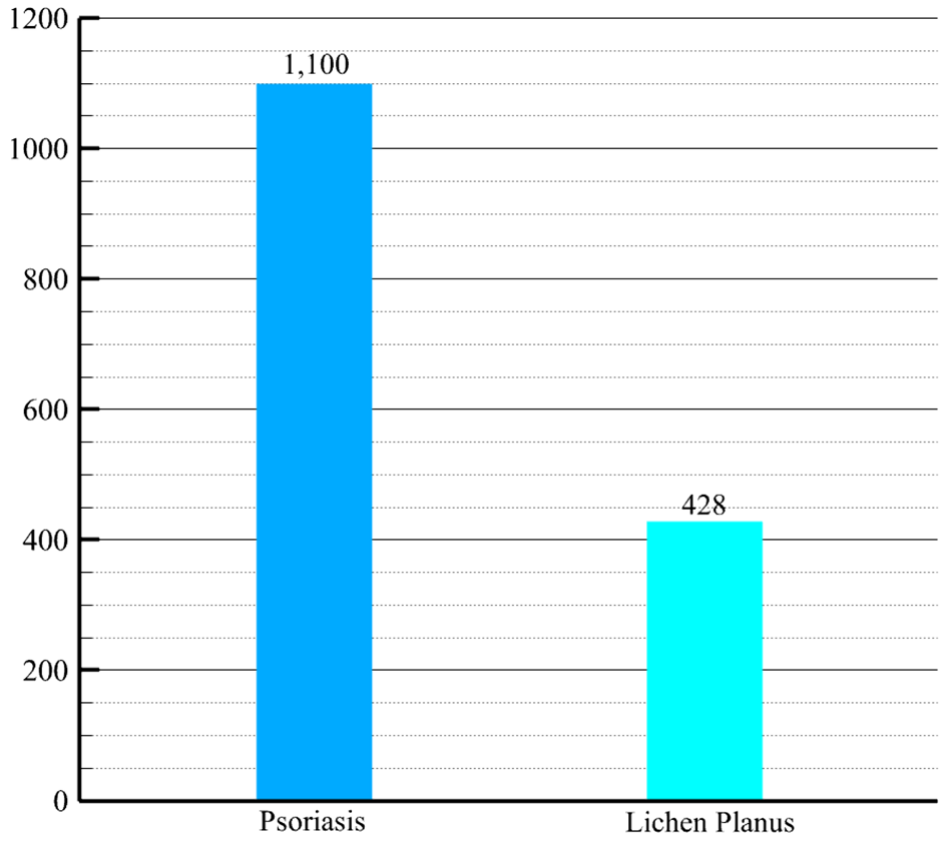
Class Imbalance in the raw dataset.

From the expansive DermNet dataset^25^, a publicly accessible repository of substantial proportions, encompassing over 23,000 images spanning in excess of 20 distinctive skin conditions, a subset comprising precisely 1,528 images was acquired. Despite the dissimilarity in their physical dimensions, the images demonstrated harmonization in terms of resolution, exhibiting a consistent horizontal and vertical resolution of 96 dots per inch (dpi). Furthermore, all images adhered to a standardized bit depth of 24, signifying the uniformity of color information within the pictorial representations. It is imperative to note that the aforementioned dataset was meticulously compiled by DermNet, with the consent of patients specifically diagnosed with either Psoriasis or Lichen planus skin conditions. Additionally, the dataset exhibited a conspicuous disparity in the distribution of its constituent categories, wherein Psoriasis instances predominated significantly, while Lichen planus representations accounted for less than one-third of the total data. This evident class imbalance is visually depicted in Fig. 1. Specifically, the Psoriasis category consisted of 1,100 available pictures, whereas the Lichen planus class comprised a smaller subset of 428 images. For the purpose of the current study, these respective categories were systematically labeled as 0 and 1, correspondingly.

**Figure 2:**
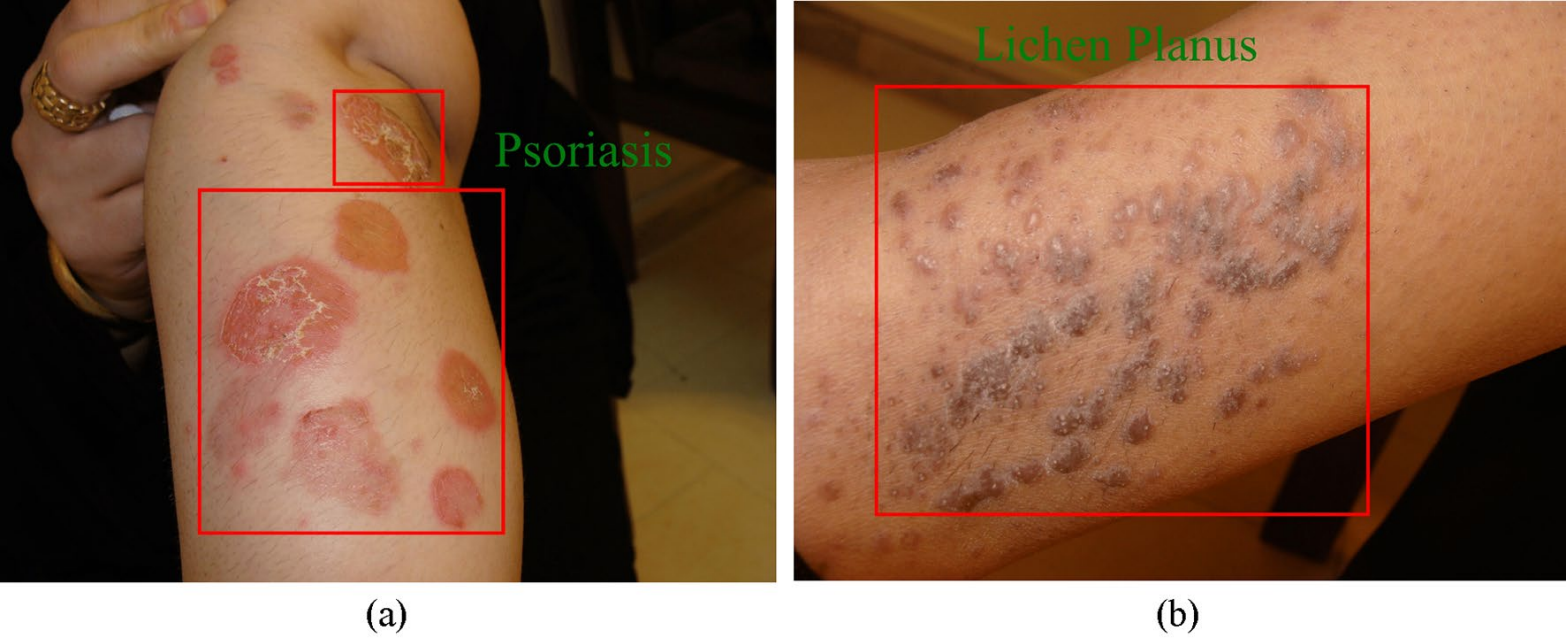
Samples of two skin disease class: (**a**) sample of Psoriasis disease from the dataset, (**b**) sample of Lichen planus disease from clinical research and development center, Semnan University of Medical Sciences.

Figure 2 showcases representative samples from the Psoriasis (2.a) and Lichen planus (2.b) categories, providing visual illustrations of the skin conditions under investigation. Notably, the afflicted regions on the skin are distinctly highlighted and delineated by means of a red rectangle, effectively demarcating the problematic areas of interest.

### Class balancing and data augmentation

**Figure 3:**
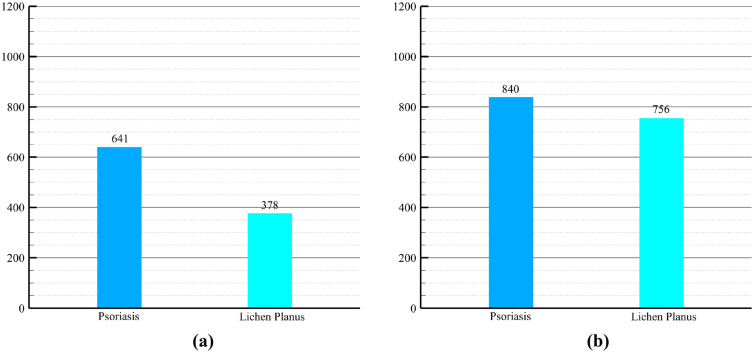
(**a**) Dataset without class balancing, (**b**) dataset with class balancing.

As elucidated in "Experimental Setup" section, the severity of class imbalance necessitated the adoption of class balancing techniques, considering its substantial impact on the false negative rates inherent in the eventual outcomes. However, an equally pertinent concern revolved around the usability of the collected data, whereby numerous images had to be excluded from the dataset due to issues pertaining to clarity, quality, and overall suitability for the present study. Consequently, the total number of images within the dataset witnessed a reduction. Nevertheless, this curation process led to a noteworthy narrowing of the gap between the classes, as the Lichen planus category contained a considerably higher proportion of usable data. Thereby, without resorting to any augmentation or balancing procedures, the dataset consisted of 641 images representing the psoriasis class and 378 images representing the lichen planus class. The crucial step of class balancing was ultimately implemented using a standard technique involving both oversampling and undersampling. This entailed duplicating certain images within the dataset, with a deliberate focus on undersampling the Psoriasis class while oversampling the Lichen planus class, effectively mitigating the disparity between the two categories. To ensure a reduction in potential information loss and biased sampling, the selection of images was conducted randomly. The resulting dataset, depicted in Fig. 3, showcases a visual representation of the dataset both before and after the application of class balancing techniques.

Considering the reduced size of the new dataset, it was determined that data augmentation would be advantageous for the study. Accordingly, the dataset was augmented by approximately 15 percent. The augmentation process involved the random selection of data and subsequent manipulation utilizing various techniques. These techniques included rotation and distortion of the images, with rotations performed at random degrees ranging from 0 to 40 degrees, and distortions applied along an axis within the range of 0–0.2 degrees. Additionally, image flipping along different axes and adjustments to brightness were implemented. The brightness adjustments were randomly selected within a range of 50–150% of the original brightness level. Moreover, zooming techniques were employed, allowing for magnification of the images up to 20% of their original size. For reference, Table 1 presents a comprehensive list of the selected augmentations employed in this process, accompanied by brief descriptions for each augmentation technique.

**Table 1:** List of selected augmentation with arguments and their description.

S. no.	Augmentation arguments	Description
1	rotation_range = 40	Rotates the images with a random angle between 0 and 40 degrees
2	shear_range = 0.2	Image will be distorted along an axis between 0 and 0.2 degrees
3	zoom_range = 0.2	Magnifies the image randomly between 0 and 20% of the image
4	horizontal_flip = True	Randomly flip images horizontally
5	vertical_flip = True	Randomly flip images vertically
6	brightness_range = (0.5, 1.5)	Shifting the brightness randomly between 50 and 150% of brightness

**Figure 4:**
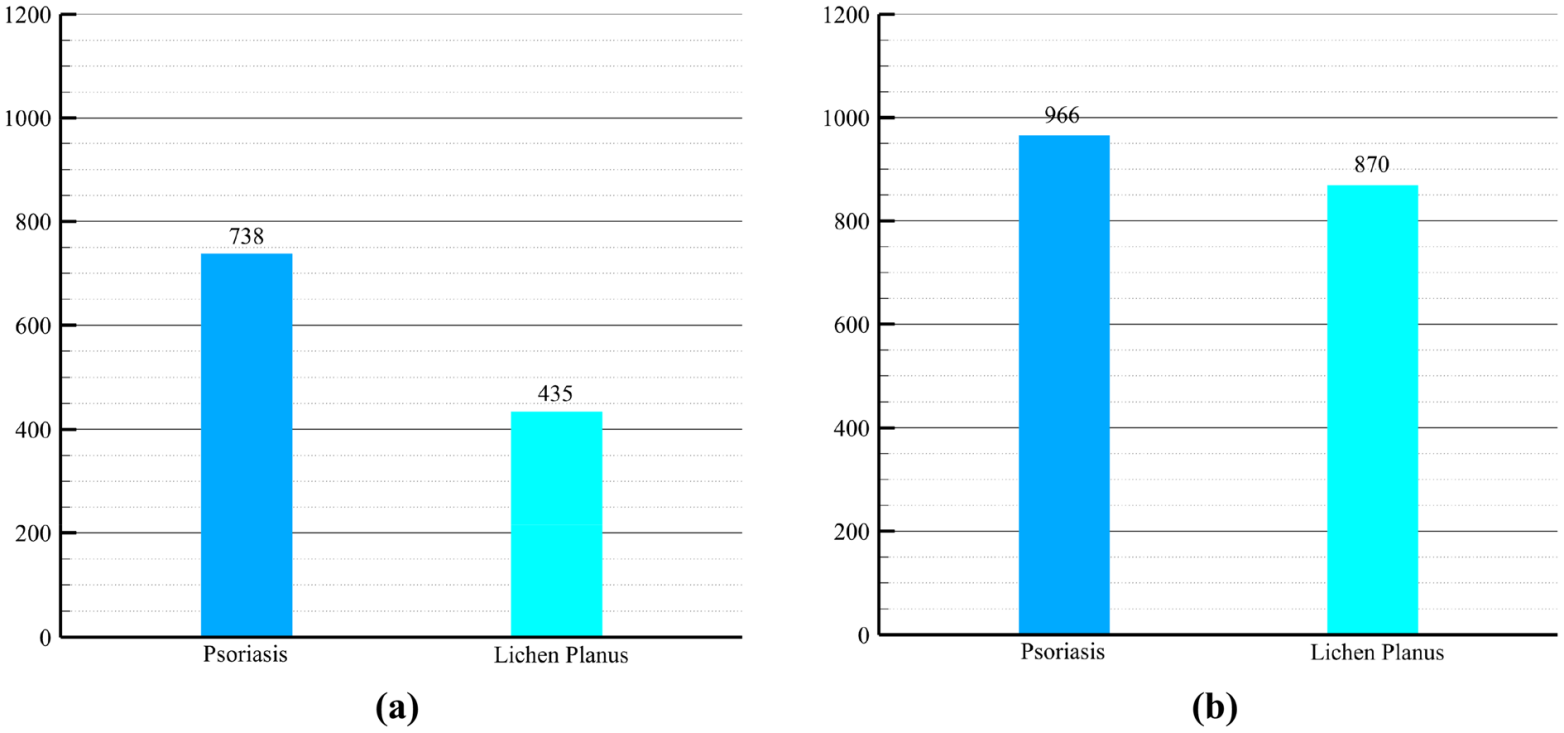
(**a**) Dataset without class balancing but with data augmentation, (**b**) dataset with class balancing and data augmentation.

The previous dataset, characterized by class imbalance, was also subjected to augmentation to facilitate its utilization. Figure 4 serves as a visual representation, illustrating the augmentation process applied to the first two datasets.

### Data pre-processing

**Figure 5:**
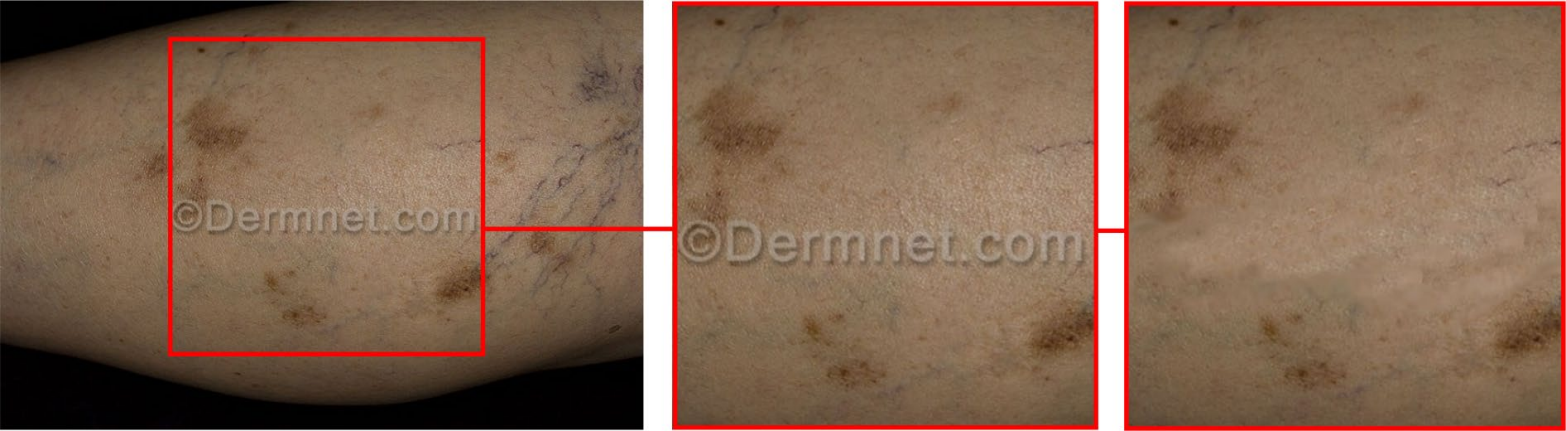
Before and after of an image through data pre-processing transformation.

To ensure the integrity of the input data and optimize the final results, pre-processing steps were employed. Firstly, a significant concern was the presence of images in the dataset that contained additional objects alongside the skin region of interest. To address this, a meticulous manual cropping process was undertaken for each file individually, aligning them at the appropriate angles to isolate the desired skin area accurately. Secondly, numerous files in the dataset were marred by watermarks, which needed to be faded out while maintaining data integrity, as the original unlabeled data was no longer available. This watermark removal process was conducted with utmost precision to avoid any damage to the underlying information. Lastly, a notable disparity was observed in the width and height of the files, rendering them incompatible with the ResNet-50 model. Consequently, resizing of the files became necessary to achieve a consistent size. The final size of the pre-processed data was established as 224 pixels × 224 pixels, ensuring compatibility with the ResNet-50 model. It is important to note that the resizing was performed conservatively, with due care taken to preserve the original proportions and shapes of the images. Figure 5 provides an exemplar of an image from the dataset after undergoing the aforementioned transformations.

### ResNet-50

**Figure 6:**
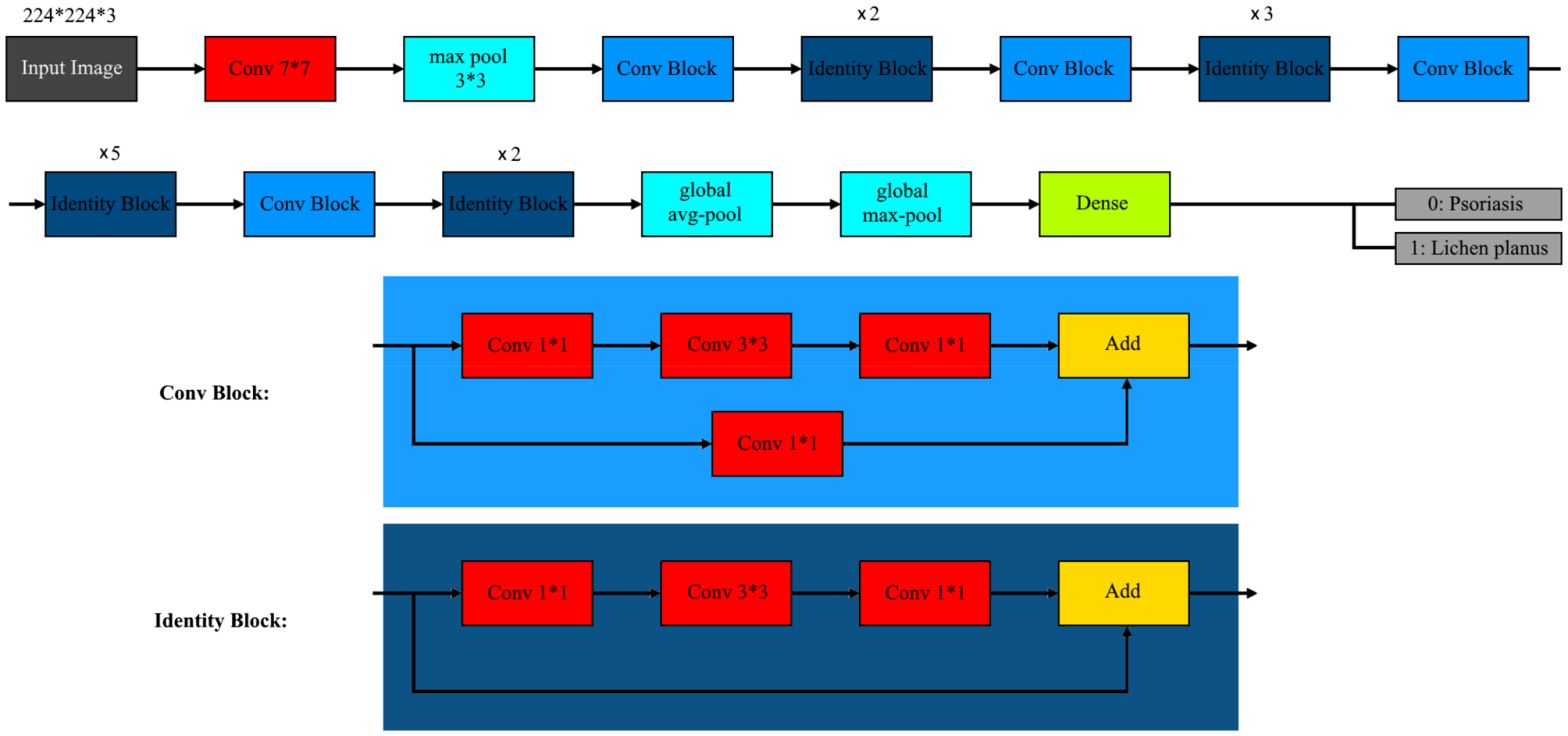
ResNet-50 architecture.

This section provides a detailed description of the proposed ResNet-50 architecture, which was achieved by considering the original ResNet-50 model available in the Keras library. The selection of ResNet-50 for our study was based on several factors that contribute to its suitability for the problem at hand. Firstly, ResNet-50 is a well-established convolutional neural network architecture known for its depth and performance in image classification tasks. Its deeper architecture allows for more complex feature extraction, which is crucial for accurately distinguishing between subtle differences in skin diseases, such as Psoriasis and Lichen Planus. Additionally, ResNet-50 has been widely adopted and extensively studied in the field of medical image analysis, including dermatology, demonstrating strong performance in various disease classification tasks^21,26^. The architecture of the proposed ResNet-50 model is depicted in Fig. 6. The input to the first layer of the model consists of images with dimensions of 224 × 224 × 3, representing height, width, and RGB color channels, respectively. Before the activation, a global max pooling layer and a dense layer were added to the model. The purpose of the max pooling layer is to reduce the dimensionality of the input, thereby decreasing the number of parameters, reducing training time, and mitigating the risk of overfitting. The dense layer performs the classification task based on the output of the preceding convolutional layers. It is worth mentioning that the original ResNet-50 model architecture, which forms the foundation of the proposed model, is readily available in the Keras library. The inclusion of the global max pooling and dense layers constitutes the modifications introduced in the proposed ResNet-50 architecture^26–29^. The performance of the model was notably influenced by manual fine-tuning of parameters and meticulous control over the learning process. In the compile stage of the model, the Adam optimizer^30^ was selected. This optimizer effectively manages the exponential decay average during the training process. To optimize the learning process, the learning rate parameter was dynamically configured based on the number of epochs. Fine-tuning the learning rate is crucial for achieving optimal performance. Through multiple trial and error experiments, it was determined that setting the number of epochs to 20 and the batch size to 32 yielded the best results. A comprehensive summary of the trial and error experiments is presented in Table 2. The parameters used for the ResNet-50 model are summarized within Table 3. The ResNet-50 model, as implemented, consists of a total of 25,636,712 parameters^31^. These parameters contribute to the overall complexity and capacity of the model, enabling it to capture intricate patterns and representations within the data.

**Table 2:** Results from class balancing and data augmentation (random splitting) with different epochs and batch sizes.

Epochs	Accuracy	Batch size	Accuracy
10	0.8670	10	0.8553
20*	0.8907*	20	0.8437
50	0.8786	32*	0.8907*
100	0.8670	60	0.8670
150	0.8437	100	0.8786

*denotes the best accuracy.

**Table 3:** Parameters used for the ResNet-50 model.

Optimizer	Epochs	Batch size
“Adam”	20	32

**Figure 7:**
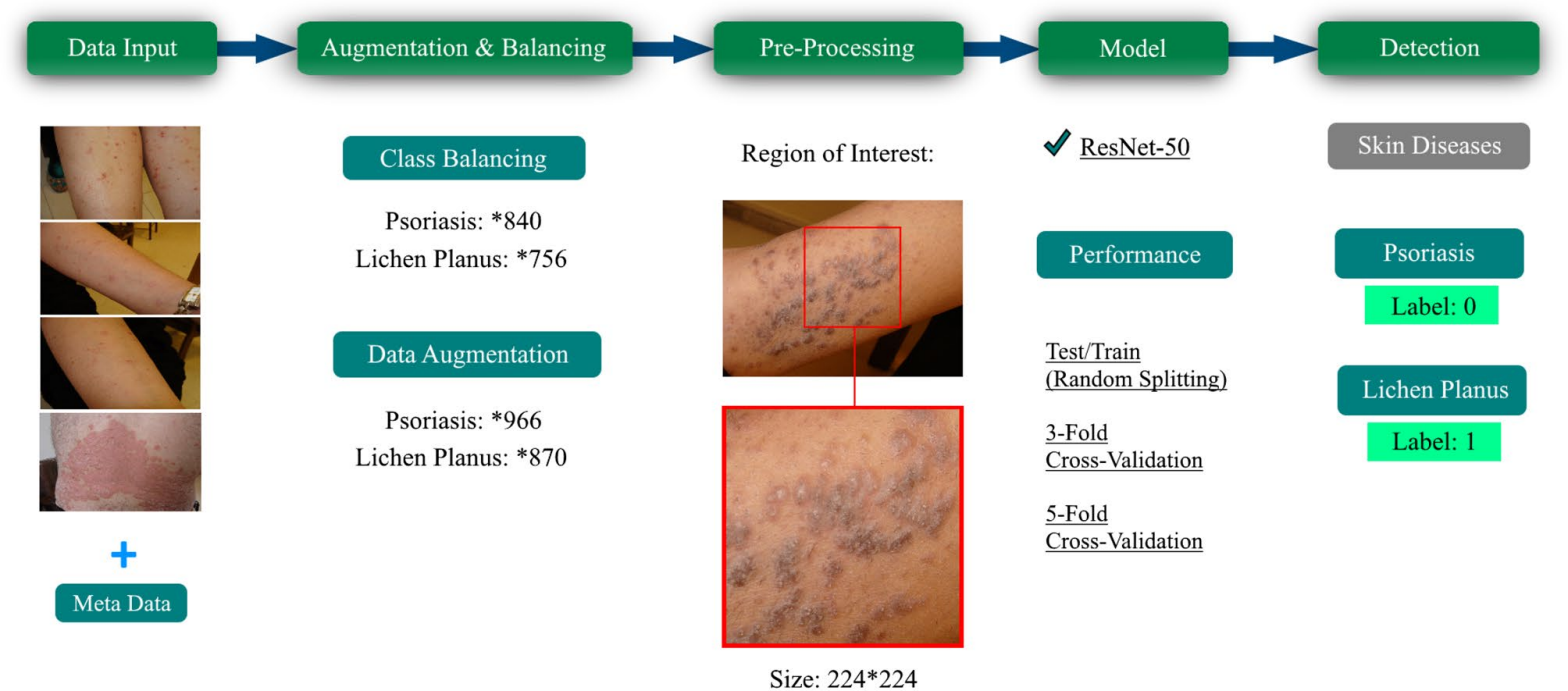
The proposed methodology in a block diagram.

Figure 7 showcases the comprehensive block diagram of the proposed work, delineated into five distinct sections. The first section involves the initial step of inserting images into the process and combining them with accompanying metadata. This integration of visual and non-visual data enables a holistic approach to data analysis. The second section encompasses augmentation and balancing techniques, wherein the gathered data is both balanced by class and augmented, resulting in the generation of multiple datasets. This process enhances the diversity and representation of the data, improving the model's robustness and performance. In the third section, the images undergo resizing and the selection of the region of interest. This step ensures consistency in the dimensions of the images and focuses on the specific areas relevant to the skin diseases under examination. The fourth section involves the insertion of the pre-processed data into the proposed ResNet-50 model. This step utilizes the modified architecture to extract meaningful features and classify the skin diseases effectively. Finally, in the fifth section, the performance of the model is evaluated through two approaches: K-fold cross-validation and random train-test splitting. These techniques assess the model's ability to accurately classify Psoriasis and Lichen planus skin diseases, providing insights into its overall performance and efficacy.

## Experimental results

This section introduces the experimental setup employed for the analysis and evaluation of the proposed model.

### Experimental setup

The trials were conducted using Jupyter Notebook^32^ with Python 3.8 as the interpreter. The experimental setup utilized a computing device with the following specifications: an Intel Core i7-7700HQ processor, an NVIDIA GeForce GTX 1060 Mobile GPU, 16GB RAM, and 1280 CUDA cores. The proposed ResNet model was implemented using Keras 2.8.0, built on the TensorFlow framework. Cross-validation methods and test/train splits were employed for the validation process of the model, ensuring robust evaluation and reliable performance assessment.

### Train/test split

**Figure 8:**
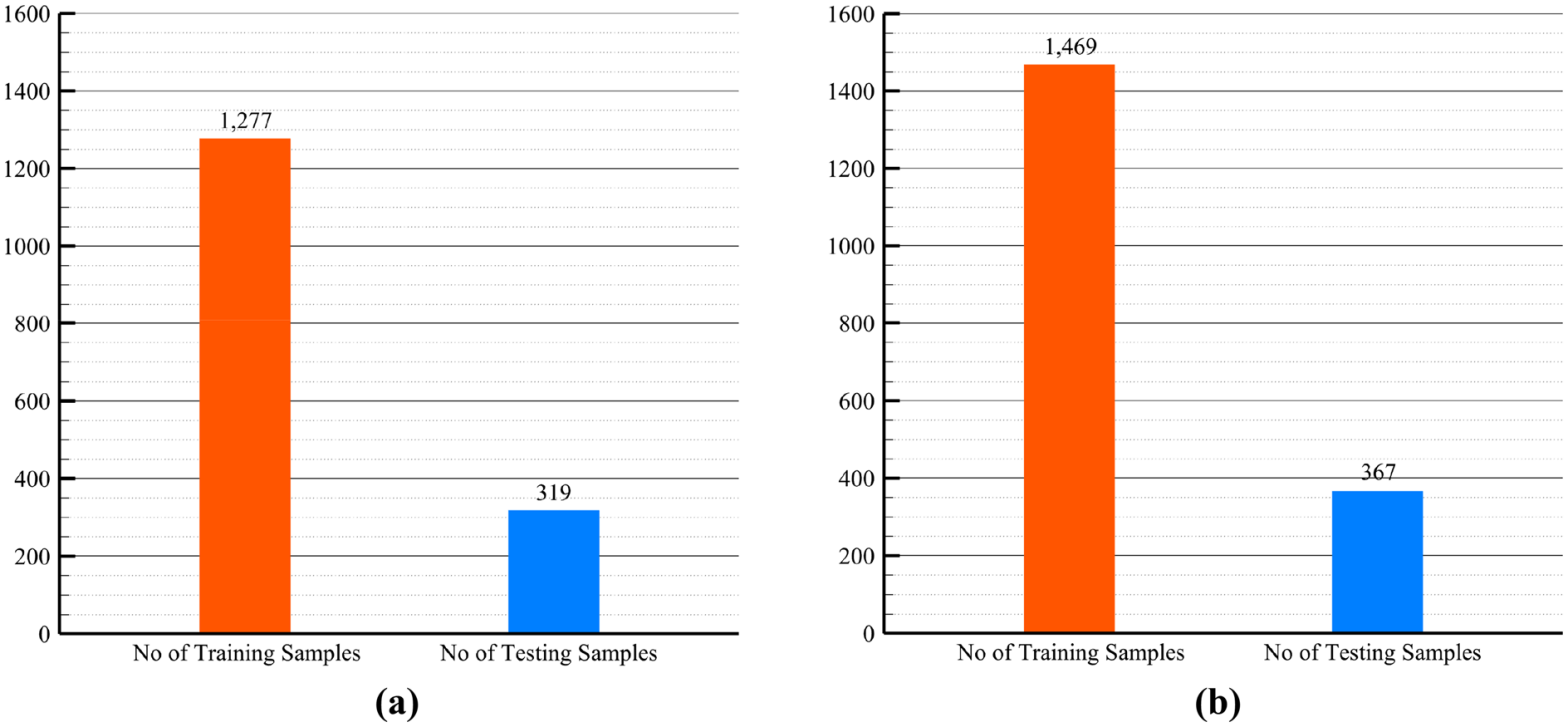
The train/test split division: (**a**) 1277 samples in train set and 319 in test set for the dataset without data augmentation, (**b**) 1469 samples in train set and 367 in test set for the dataset with data augmentation.

In the two approaches, considering both with and without data augmentation, the ResNet model was validated using a train and test split technique. Approximately one-fifth of each dataset was allocated for testing, while the remaining 80% was utilized for training the model. The precise breakdown of the train/test split is presented in Fig. 8, providing a comprehensive view of the distribution of data between the training and testing phases.

### K-fold cross-validation

The ResNet model was also validated using K-fold cross-validation techniques. In this process, the dataset is randomly divided into K different folds or groups. The model is then validated K times, with each iteration using one fold for validation while the remaining folds are used for training the model. To address potential bias introduced by multiple images from the same patient, cross-validation was conducted by stratifying the folds based on augmented patient data. This ensured that each fold contained images from different patients, thereby enhancing the robustness of the evaluation process. The final accuracy is calculated as the mean of all the accuracies achieved during this process. Similarly, the final results are calculated as the mean of all the validation results. In this experiment, the value of K was configured differently for each dataset variation. For the class balancing with data augmentation dataset, K was set to 3 and k was set to 5. This means that the model was validated 5 times, with each validation using 3 randomly selected folds for training and onefold for validation. For the dataset without class balancing, K was set to 5, and for the dataset without class balancing with data augmentation, K was also set to 5. By utilizing K-fold cross-validation, a more robust evaluation of the model's performance is achieved, as it takes into account variations in the dataset and provides a more accurate estimate of the model's generalization capabilities.

### Performance metrics

For the evaluation of the model's performance, several metrics were measured including accuracy, sensitivity, precision, F1-score, specificity, area under the curve (AUC), receiver operating characteristic (ROC) curve. The results for these metrics are presented in Table 4. Accuracy is a metric that measures the proportion of correctly predicted data points out of all the data points. It is calculated by dividing the number of correctly predicted data points by the total number of data points. Accuracy is described in Eq. (1).

1 $$Accuracy = \frac{TP + TN}{{TP + TN + FP + FN}}$$

where TP (True Positive) signifies the count of accurately predicted positive instances, while TN (True Negative) denotes the count of precisely predicted negative instances. In contrast, FP (False Positive) symbolizes the count of erroneously predicted positive instances, and FN (False Negative) embodies the count of inaccurately predicted negative instances. These metrics stand as valuable indicators, imparting insights into the operational proficiency of such models.

**Table 4:** Performance Metrics of model ResNet-50 convolutional neural network (CNN).

Evaluation metrics of ResNet-50 CNN				
	Multi classes			
Approaches	Evaluation	Psoriasis	Lichen planus	Average
Without class balancing and data augmentation (fivefold cross-validation)	Precision	0.7735	0.6570	0.7152
	Sensitivity	0.5922	0.8667	0.7048
	F1-Score	0.6693	0.7287	0.7048
	Specificity	0.7342	0.7342	0.7342
	Accuracy			0.7767
	AUC	0.7590	0.7590	0.7590
Without class balancing but data augmentation (fivefold cross-validation)	Precision	0.7869	0.7108	0.7488
	Sensitivity	0.6000	0.8587	0.7293
	F1-Score	0.6796	0.7773	0.7284
	Specificity	0.7632	0.7632	0.7632
	Accuracy			0.7632
	AUC	0.8807	0.8807	0.8807
Class balancing and without data augmentation (random splitting)	Precision	0.8483	0.7640	0.8061
	Sensitivity	0.8483	0.7640	0.8061
	F1-Score	0.8482	0.7639	0.8061
	Specificity	0.8039	0.8039	0.8039
	Accuracy			0.8039
	AUC	0.9071	0.9071	0.9071
Class balancing and data augmentation (random splitting)	Precision	0.9222	0.8602	0.8912
	Sensitivity	0.9195	0.8646	0.8920
	F1-Score	0.9208	0.8623	0.8916
	Specificity	0.8907	0.8907	0.8907
	Accuracy			0.8907
	AUC	0.9678	0.9678	0.9678
Class balancing and data augmentation (threefold cross-validation)	Precision	0.8868	0.8173	0.8778
	Sensitivity	0.8851	0.8178	0.8514
	F1-Score	0.8859	0.8175	0.8517
	Specificity	0.8496	0.8496	0.8496
	Accuracy			0.8496
	AUC	0.9163	0.9163	0.9163
Class balancing and data augmentation (fivefold cross-validation)	Precision	0.9099	0.8207	0.8653
	Sensitivity	0.9103	0.8190	0.8646
	F1-Score	0.9101	0.8198	0.8649
	Specificity	0.8626	0.8626	0.8626
	Accuracy			0.8635
	AUC	0.9298	0.9298	0.9298

Sensitivity, also known as true positive rate or recall, measures the proportion of actual positive cases that are correctly identified by the model. Sensitivity is described in Eq. (2).

2 $${\text{Sensitivity}} = \frac{TP}{{TP + FN}}$$

Precision represents the proportion of correctly predicted positive cases out of all the predicted positive cases. It provides insight into the model's ability to minimize false positive predictions. This metric is described in Eq. (3).

3 $$Precision = \frac{TP}{{TP + FP}}$$

The F1-score is the harmonic mean of precision and sensitivity. It provides a balanced measure of the model's performance by taking into account both precision and sensitivity, and is, described in Eq. (4).

4 $$F1 score = 2 \times \frac{Precision \times Recall}{{Precision + Recall}}$$

Specificity measures the proportion of actual negative cases that are correctly identified by the model. Equation (5) describes this metric.

5 $$Specificity = \frac{TN}{{TN + FP}}$$

The ROC (Receiver Operating Characteristic) curve is a metric used to evaluate binary classification problems. It plots the true positive rate (TPR) against the false positive rate (FPR) at various classification thresholds on a probability curve. The AUC (Area Under the Curve) is a summary measure of the ROC curve. It quantifies the classifier's ability to distinguish between different classes. The AUC is calculated by integrating the area under the ROC curve. The formulation for AUC is elucidated in Eq. (6).

6 $$AUC = \int True \,Positive\, Rated\left( {False\, Positive\, Rate} \right)$$

### Prediction performance of proposed ResNet-50

The proposed ResNet-50 model was compiled using categorical cross-entropy as the loss function parameter and Adam optimizer^30^ as the optimizer parameter. The batch size for training the model was set to 32, and the model was trained for a total of 20 epochs. The performance of the model was evaluated using various approaches, which are detailed in the study. The results of these evaluations, including the test loss and test accuracy, are presented in Table 5. These metrics provide insights into the performance of the model on the test dataset, indicating the effectiveness of the proposed ResNet-50 model in classifying the Psoriasis and Lichen planus skin diseases.

**Table 5:** The ResNet-50 CNN test loss and test accuracy.

ResNet-50 CNN model tested approaches	Test loss	Test accuracy
Without class balancing and data augmentation (fivefold cross-validation)	1.0784	0.7767
Without class balancing but data augmentation (fivefold cross-validation)	0.7262	0.7632
Class balancing and without data augmentation (random splitting)	0.8078	0.8039
Class balancing and data augmentation (random splitting)	0.3716	0.8907
Class balancing and data augmentation (threefold cross-validation)	0.6489	0.8496
Class balancing and data augmentation (fivefold cross-validation)	0.5928	0.8635

Among the different approaches evaluated, the class balancing and data augmentation with random splitting technique achieved the best results in terms of loss, with a value of 0.3716. This approach also demonstrated the highest accuracy of 89.07%. However, it is worth noting that the class balancing and data augmentation with fivefold cross-validation approach achieved a slightly lower accuracy of 86.35%, but it may provide a more robust evaluation due to the use of cross-validation. The performance of each approach is thoroughly documented in Table 5, providing a comprehensive overview of the metrics for each technique. It is evident that class balancing has had a substantial impact on the performance of the model. The approaches without class balancing showed the lowest performance, regardless of the augmentation technique used. Even with minimal augmentation, a modest increase in accuracy was observed when the datasets were balanced.

Table 5 provides a more detailed description of the performance metrics for the proposed model. In general, the metrics, except for AUC, averaged out close to the accuracy metric with minimal discrepancies across the different approaches. AUC, on the other hand, tended to be relatively higher in each scenario. This consistent pattern can be observed throughout all of the evaluated approaches. These findings highlight the significance of class balancing and data augmentation techniques in improving the performance of the proposed ResNet-50 model for the classification of Psoriasis and Lichen planus skin diseases.

## Discussion

This study presents a comprehensive investigation into the automated classification and identification of Psoriasis and Lichen planus skin diseases using a Residual Network with 50 layers (ResNet-50) convolutional neural network (CNN) based on deep learning techniques. The study also provides an overview of relevant research in this field. The performance of the ResNet-50 CNN model was thoroughly evaluated and compared under various conditions, including the implementation of data augmentation and class balancing techniques. The results demonstrated that without class balancing, the overall accuracy for identifying the cutaneous diseases fell short of the 80% threshold. However, with the application of data augmentation and class balancing, the accuracy significantly improved. In terms of data augmentation, the difference in accuracies between the cases with and without implementation was not substantial, ranging from 86.35 to 89.07%. Nevertheless, the approach that combined class balancing and data augmentation yielded the highest accuracy among all the evaluated approaches. This study highlights the potential and effectiveness of the ResNet-50 CNN model in accurately detecting and classifying Psoriasis and Lichen planus skin diseases. The findings emphasize the importance of considering data augmentation and class balancing techniques to enhance the accuracy and efficiency of such automated systems for dermatological diagnoses.

**Figure 9:**
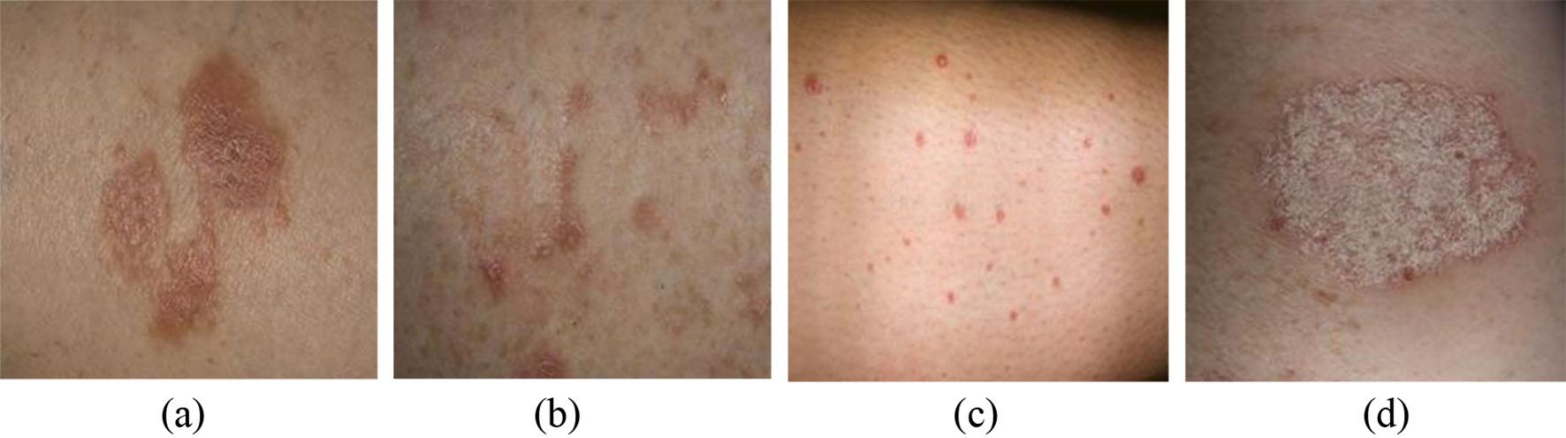
Examples of correctly diagnosed and misdiagnosed images. (**a**) Misdiagnosed Lichen planus, (**b**) correctly diagnosed Lichen planus, (**c**) Misdiagnosed Psoriasis, (**d**) Correctly diagnosed Psoriasis.

Figure 9 showcases examples of correctly diagnosed and misdiagnosed images, providing visual illustrations of the performance of the proposed ResNet-50 model. These images serve as representative samples to demonstrate the accuracy of the model in correctly identifying Psoriasis and Lichen planus skin diseases.

**Figure 10:**
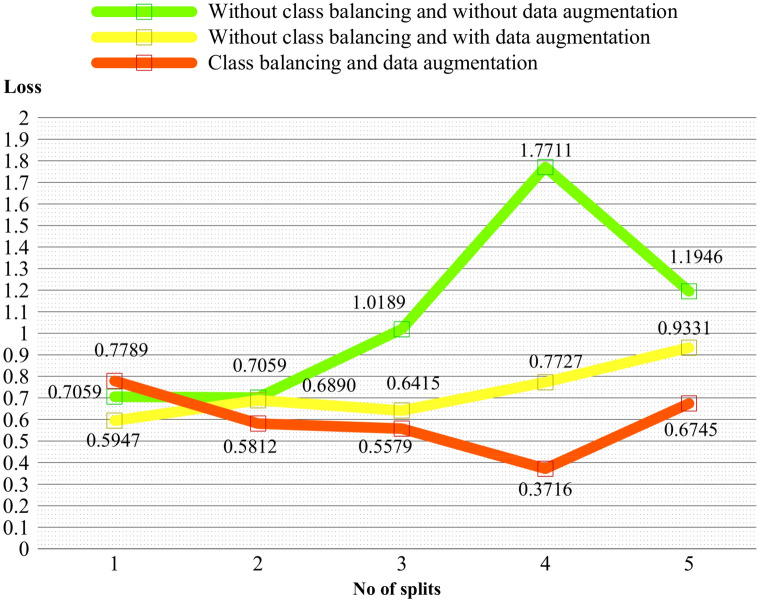
Loss comparison of class balancing and augmentation through fivefold cross validation.

In Fig. 10, the loss values for the three different K-fold cross-validation approaches are compared. The approaches include: without class balancing and without data augmentation, without class balancing and with data augmentation, and class balancing with data augmentation. The chart visualizes the loss values obtained for each fold or split in the cross-validation process. The orange line represents the recommended approach, which involves class balancing and data augmentation. This approach consistently demonstrates the lowest loss values and thus provides the best results across the different folds or splits. This comparison reinforces the effectiveness of utilizing class balancing and data augmentation techniques to improve the performance of the ResNet-50 model in accurately diagnosing Psoriasis and Lichen planus skin diseases.

**Figure 11:**
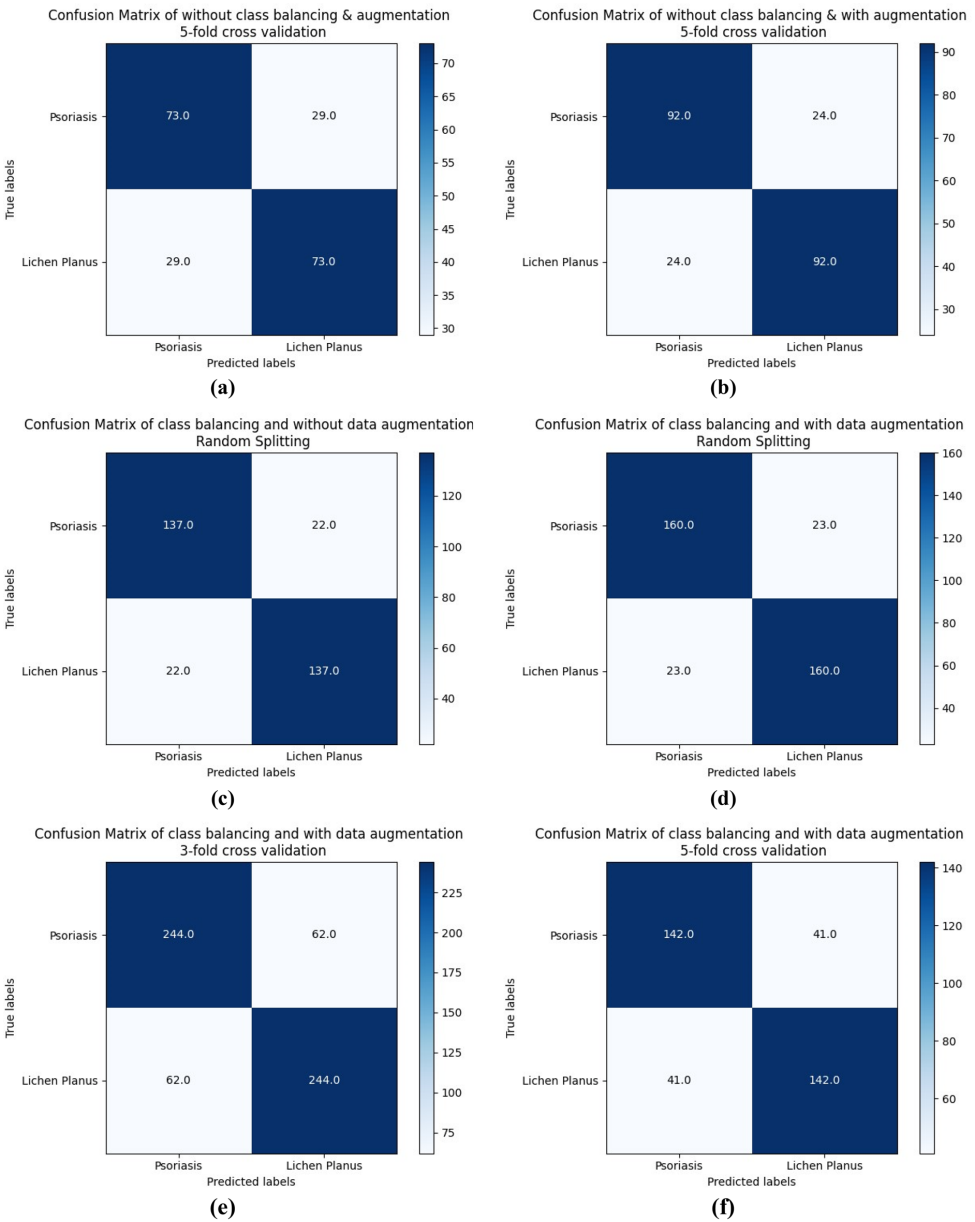
The Confusion Matrix of the ResNet-50 CNN Model. (**a**) The Confusion Matrix of fivefold cross-validation of without class balancing and augmentation. (**b**) The Confusion Matrix of fivefold cross-validation of without class balancing and with augmentation. (**c**) The Confusion Matrix of random splitting of class balancing and without augmentation. (**d**) The Confusion Matrix of random splitting of class balancing and with augmentation. (**e**) The Confusion Matrix of threefold cross-validation of class balancing and augmentation. (**f**) The Confusion Matrix of fivefold cross-validation of class balancing and augmentation.

Figure 11 presents the confusion matrix for all of the approaches, which shows the distribution of false positive, false negative, true positive, and true negative classifications for Psoriasis and Lichen planus classes. The matrix provides a visual representation of the model's performance in correctly classifying the skin diseases.

**Figure 12:**
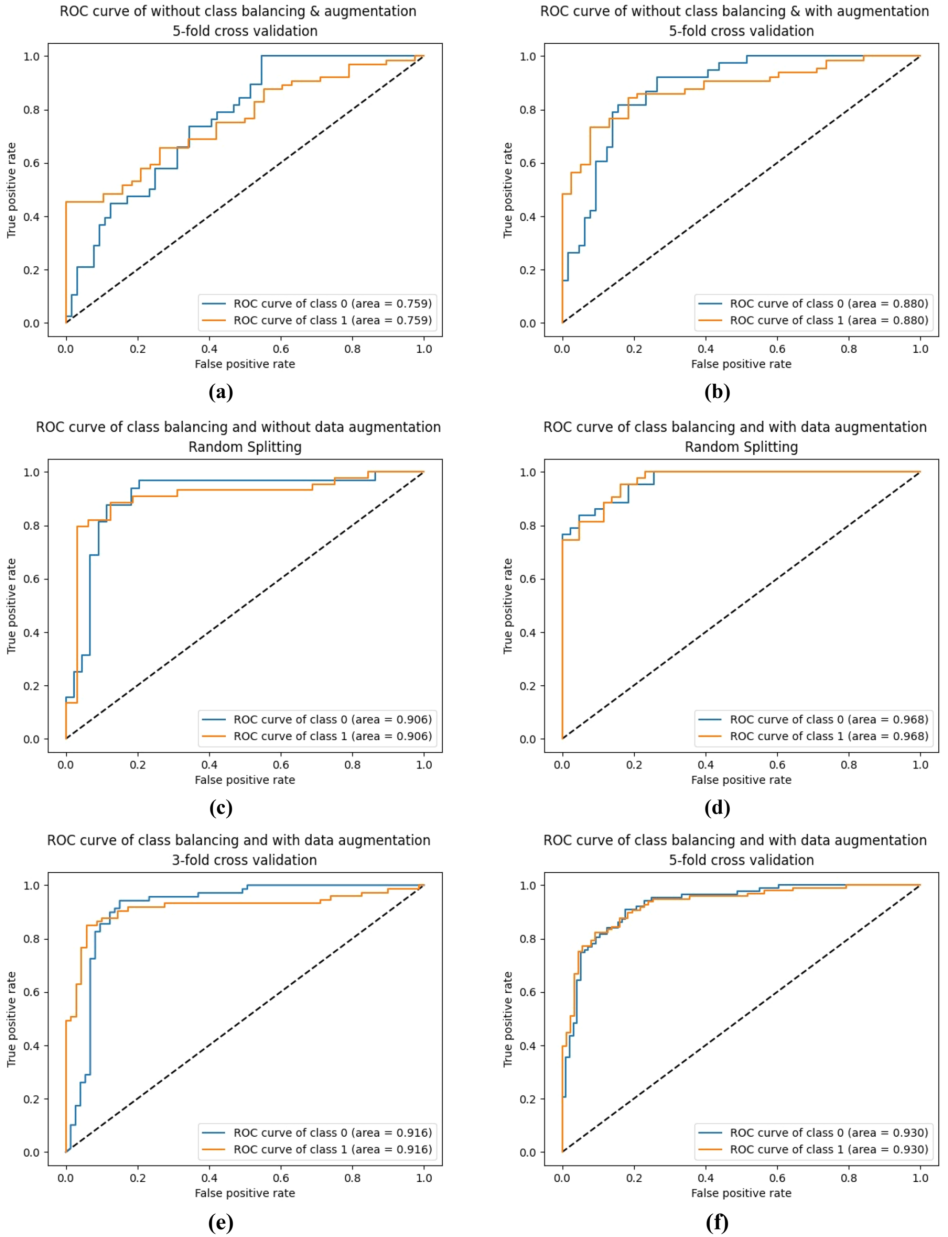
The ROC curves of the ResNet-50 CNN Model. (**a**) The ROC curves of each class for fivefold cross-validation of without class balancing and augmentation. (**b**) The ROC curves of each class for fivefold cross-validation of without class balancing and with augmentation. (**c**) The ROC curves of each class for random splitting of class balancing and without augmentation. (**d**) The ROC curves of each class for random splitting of class balancing and with augmentation. (**e**) The ROC curves of each class for threefold cross-validation of class balancing and augmentation. (**f**) The ROC curves of each class for fivefold cross-validation of class balancing and augmentation.

Figure 12 displays the receiver operating characteristic (ROC) curves for the six approaches. These curves illustrate the true positive ratio and false positive ratio at various classification thresholds. The area under the curve (AUC) is calculated to quantify the performance of each approach. The class balancing with data augmentation approach achieves an impressive AUC of 96%, indicating its high discriminatory ability in distinguishing between Psoriasis and Lichen planus skin diseases.

Table 6 provides a comparison between the current study and four other research papers that have focused on using deep learning and convolutional neural networks for the classification and detection of Psoriasis and/or Lichen planus skin diseases. In the study by Yang, Wang et al. in 2021, the performance of their approach was measured, but they did not report the class-based accuracy or the total accuracy for their method. While they achieved favorable sensitivity and specificity results, the overall accuracy of their approach remains unknown. Although the study covers multiple skin conditions, the authors have stated that the primary focus of their investigation is on Psoriasis. Furthermore, the dataset utilized consist of imaging data collected from China. These factors limit the applicability of their approach in recognition of Psoriasis vs. Lichen Planus. Zhao, Xie et al. in 2019 explored deep learning models for the design of a smart identification system based on clinical images, but their study was limited to Psoriasis disease only. Zhu, Wang et al. in 2021 constructed a framework based on deep learning and trained it on a dataset of 13,603 images, which represented the clinical environment in China. However, the accuracy reported in their study may be inflated due to the specificity of their dataset, which may not be applicable to different environments. Bajwa, Muta et al. in 2020 used the DermNet and ISIC Archive to train Deep Neural Networks for the classification of 23 skin lesions, including Psoriasis, Lichen planus, and related diseases. However, their approach did not provide conclusive differentiation between Psoriasis and Lichen planus. Gunwant et al. (2022) developed an advanced expert system utilizing the EfficientNet B-0 model to aid clinicians in identifying various cutaneous diseases. The study utilized the DermNet dataset and achieved an impressive average accuracy rate of 91.36%. However, similar to conventional methodologies, Psoriasis and Lichen were grouped together, representing a limitation in disease differentiation. Similarly, the deep learning approach proposed by Mohamed Hammad et al. (2023) focused on detecting eczema and psoriasis. Despite achieving remarkable results with an accuracy of 96.20%, precision of 96%, recall of 95.70%, and F1-score of 95.80%, the study solely concentrated on psoriasis, limiting its scope. Nieniewski et al. (2023) investigated differentiating psoriasis from other dermatoses using a small dataset and transfer learning techniques. While achieving a sensitivity of 85.33% and a precision of 82.58%, the study's exclusive focus on psoriasis represents a notable limitation, restricting its applicability to broader dermatological conditions. Additionally, variability in working conditions due to smartphone image capture adds another layer of constraint to the study's findings. It is important to recognize that the focus of our research on a unique subject makes it impractical to replicate the exact experimental conditions used in other studies. The data we employed has not been previously examined for this specific purpose, further contributing to the distinctive nature of our investigation. Additionally, it's crucial to emphasize that the purpose of comparing models is not to assert the superiority of one over another; rather, it serves to underscore the strengths inherent in each approach; These comparisons highlight the strengths and limitations of each approach and emphasize the importance of considering dataset diversity and environmental factors when evaluating the performance of deep learning models for skin disease classification.

**Table 6:** Comparison state of the art works with our proposed model.

Author, year	Model	Dataset	Target output	Evaluation				
				Accuracy	Sensitivity	Precision	Specificity	AUC
Yang et al., 2021^18^	EfficientNet-B4 (two-class CNN)	Dermoscopic images (7,033)	Mean	–	0.927	–	0.827	–
			Psoriasis	–	0.688	–	0.903	–
			Lichen Planus	–	0.669	–	0.953	–
Yang et al., 2021^18^	EfficientNet-B4 (four-class CNN)	Dermoscopic images (7,033)	Mean	–	0.889	–	0.968	–
			Psoriasis	–	0.929	–	0.952	–
			Lichen Planus	–	0.933	–	0.960	–
Zhao et al., 2019^19^	InceptionV3 (One-stage)	XiangyaDer-Pso9 (8,021)	Psoriasis	–	0.91	–	0.96	0.966
	DenseNet121 (Two-stage)			–	0.83	–	0.97	0.954
	InceptionResNetV2 (Two-stage)			–	0.95	–	0.97	0.975
	Xception (Two-stage)			–	0.93	–	0.98	0.976
	InceptionV3 (Two-stage)			–	0.92	–	0.98	0.981
Zhu et al., 2021^1^	EfficientNet-B4 (modified)	Imaging database of the Department of Dermatology (13,603)	Mean	0.948	0.934	–	0.950	–
			Psoriasis	0.886	0.920	–	0.882	–
			Lichen Planus	0.969	0.873	–	0.975	–
Bajwa et al., 2020^20^	ResNet-152, DenseNet-161, SE-ResNeXt-10, NASNet	DermNet (2,112)	Psoriasis, Lichen Planus and related diseases	–	0.8191	0.7961	0.9726	–
Gunwant et al.^21^	Efficinetnet-B0, ResNet-50	DermNet (≈2,400)	Psoriasis and Lichen Planus	0.9136 (Ave)	0.9268 (Ave)	–	–	–
Hammad et al.^23^	AlexNet, ResNet, and VGG-16	Dermoscopic images (2055)	Psoriasis		0.9570	0.96	–	–
Nieniewski et al.^24^	Support Vector Machine	Smartphone cameras pictures (150 patients)	Psoriasis	–	0.8533	0.8258	–	–
Proposed ResNet-50		DermNet (856)	Mean	0.8907	0.8920	0.8912	0.8907	0.9678
			Psoriasis		0.9195	0.9222	0.8907	0.9678
			Lichen Planus		0.8646	0.8602	0.8907	0.9678

## Limitations

The study acknowledges several limitations that should be taken into consideration. Firstly, the validation process using K-fold cross-validation could be further optimized by evaluating all folds together rather than individually. This would reduce the training burden and provide a more uniform evaluation process. Another limitation is the relatively small size of the dataset used in this study compared to other similar researches. Increasing the dataset size would enhance the robustness and generalizability of the model. Collecting a larger dataset should be considered in future studies to improve the accuracy and reliability of the results. Furthermore, during the data collection process, it was observed that there was a significant difference in the availability of Psoriasis and Lichen planus images. This imbalance between the classes may have introduced biases in the model towards the class with more data, which in this case is Psoriasis. Although class balancing techniques were applied, there was still a noticeable disparity between the two classes. To mitigate this issue, alternative solutions such as class weighting or other sampling techniques could be explored. Additionally, the authors recommend collecting more data specifically for the minority class (Lichen planus) to address this imbalance. These limitations highlight areas for improvement in future studies, including optimizing the validation process, increasing the dataset size, and addressing class imbalance to ensure more accurate and unbiased results.

## Conclusions

This study presents an efficient and automated computer-based system for the identification and classification of Psoriasis and Lichen planus cutaneous diseases. The proposed approach utilizes a Residual Network with 50 layers Convolutional Neural Network (CNN) architecture. By employing advanced techniques such as data augmentation and class balancing, the dataset's diversity is increased and the class imbalance is mitigated. The validation of the model is performed using various techniques including fivefold cross-validation, threefold cross-validation, and random splitting. These methods ensure the reliability and generalizability of the results. The ResNet-50 CNN model demonstrates high accuracy, sensitivity, precision, specificity, and area under the curve (AUC) values. The achieved accuracy of 89.07% is remarkable considering the relatively small dataset used in this study. The sensitivity and precision values further validate the effectiveness of the proposed approach in correctly identifying and classifying the skin diseases. Furthermore, the performance metrics such as specificity and AUC indicate the model's ability to differentiate between the classes with high accuracy. Moreover, due to its exceptional accuracy, the recommendation of the proposed ResNet-50 model for the classification of these two diseases is warranted. It is noteworthy that despite the smaller dataset size compared to other studies, the results obtained in this research are comparable and even surpass those achieved with much larger datasets. While this study employs a classic approach to address the classification problem, the novelty arises from the scarcity of research specifically focusing on these two lesions without the presence of other classes. Moreover, the model demonstrated exceptional performance criteria, further underscoring the significance of the findings. To the authors' knowledge, this study presents a unique and effective automated deep learning computer-based system specifically designed for the classification and identification of Psoriasis and Lichen planus skin diseases. The proposed approach demonstrates promising results and highlights the potential of deep learning techniques in the field of dermatology.

## Data Availability

The datasets used and analyzed during the current study available from the corresponding author on reasonable request.
